# Development of a Method to Detect *Mycobacterium paratuberculosis* in the Blood of Farmed Deer Using Actiphage® Rapid

**DOI:** 10.3389/fvets.2021.665697

**Published:** 2021-07-29

**Authors:** Anton Kubala, Tania M. Perehinec, Catherine Evans, Andrea Pirovano, Benjamin M. C. Swift, Catherine E. D. Rees

**Affiliations:** ^1^School of Biosciences, University of Nottingham, Loughborough, United Kingdom; ^2^PBD Biotech Ltd., Link House, Elm Farm Park, Thurston, United Kingdom; ^3^Pathobiology and Population Sciences, The Royal Veterinary College, Hatfield, United Kingdom

**Keywords:** *Mycobacterium paratuberculosis*, Actiphage, deer, bacteriophage, qPCR

## Abstract

*Mycobacterium avium* subsp paratuberculosis (MAP) is the causative agent of Johne's disease, which is an economically and clinically relevant pathogen for commercial deer production. The purpose of this study was to develop a method that could be used to rapidly detect MAP infection in deer using the Actiphage Rapid blood test. This test has previously been used to detect MAP in cattle blood following the purification of buffy coat using Ficoll gradients, however this method is quite laborious and costly. The purpose of this study was to develop a simpler method of blood preparation that was also compatible with deer blood and the Actiphage test. Initially differential lysis of RBCs using Ammonium Chloride-Potassium (ACK) blood lysis buffer was compared with the Ficoll gradient centrifugation method using cattle blood samples for compatibility with the Actiphage reagents, and it was found that the simpler ACK method did not have an impact on the Actiphage test reagents, producing an equivalent sensitivity for detection of low levels of MAP. When the two methods were compared using clinical blood samples from farmed deer, the ACK lysis method resulted in a cleaner sample. When a blinded test of 132 animals from 4 different production groups was carried out, the majority of the positive test results were found to be from animals in just one group, with a small number identified in a second group. The test results were found to be reproducible when a small set of positive animals were tested again 1 month after their initial testing. Finally a set of negative animals which had been previously screened using an ELISA test, all animals gave a negative Actiphage result. This study shows that this improved sample preparation method and Actiphage blood testing can be used to test blood samples from deer, and the full diagnostic potential of the method can now be evaluated.

## Introduction

*Mycobacterium avium* subsp. *paratuberculosis* (MAP) is a slow growing acid-fast bacterium that is the causative agent of Johne's disease in a range of farmed ruminants including cattle, sheep, goats and deer (1). Johne's disease is a chronic inflammation (granulomatous enteritis of the intestine primarily affecting the jejunum and ileum) resulting in inhibition of nutrient absorption and leading to chronic wasting of the animal resulting in reduced meat yields, reduced fertility and premature death of the animals (2). Although good estimates of the economic impact of Johne's disease for commercial deer farming are not available, it is known that the disease does pose a significant cost to other farmed species susceptible to Johne's disease (3, 4).

In farmed, captive and free-living deer, diarrhoea, loss of weight and body condition are clinical signs of disease (5). Two clinical syndromes have been described in red deer: sporadic disease with low morbidity and high mortality in adult populations, and severe outbreaks in young deer (8–15 months old) resulting in both high morbidity and high mortality (2). In infected animals, MAP is increasingly shed in faeces as the disease progresses [see (6)]. Contaminated faeces then acts as a source of transmission within herds by the faecal-oral route (7). MAP can also be transmitted through ingestion of colostrum from infected dams or *in utero via* the placenta and this is reported to be very high in both symptomatic and asymptomatic red deer (8, 9). Therefore, for control of the disease in farmed deer, it is important to have accurate tests that can detect infection at an early stage of infection (4, 10).

Culture-based diagnostic approaches that can be used for other pathogenic organisms are not appropriate for mycobacterial pathogens due to their very slow *in vitro* growth rates. Culture-based tests require from 4 weeks (automated liquid culture) to 20 weeks [culture on solid media; (11)]. The main diagnostic tool currently employed for the rapid detection of Johne's disease is an antibody ELISA which detects the presence of MAP-specific antibodies in either blood or milk produced by the animal in response to infection. In cattle populations, the MAP ELISA is often used as a front line test due to its availability and fast sample throughput, but its shortcomings include low sensitivity and accuracy especially during early stages of the infection (4, 6, 10). As intracellular pathogens, MAP is known to infect and replicate inside macrophages and have the ability to evade immune surveillance and signalling pathways, allowing them to persist in the intracellular environment for long periods of time before shedding becomes evident. In cattle animal's immune response to MAP is not consistent over time and antibody production is slow to develop [see (6)]. However, it has been shown that IgG ELISA tests that have been optimised for cattle samples do not retain their performance characteristics when used to test blood samples from other species (12, 13). In contrast to cattle, farmed deer do seem to raise a detectable antibody response (14), and surveys have been performed to evaluate the performance of different ELISA tests (13) but there are still limited numbers of commercial validated tests available for deer. The IgG1 ELISA for detection of paratuberculosis in deer (Paralisa™) has been developed in New Zealand and has been shown to be of value for screening deer herds to identify high shedding animals (15), although this was also not found to have a good predictive value in earlier stages of infection. Therefore, new tests that can directly detect the bacterium in the early stages of infection could be used to help implement control or eradication programmes.

Phage-based assays, such as the Actiphage® assay (PBD Biotech Ltd., UK), have been shown to be able to detect MAP in the blood of cattle (16, 17). The assay uses mycobacteriophage D29 as a lysing agent to efficiently release genomic DNA from low numbers of mycobacterial cells which can be detected by signature specific PCR assays (16, 17). In addition to using phage-based assays to detect MAP in blood as a sign of infection, we have previously shown that the appearance of detectable levels of mycobacteria in the circulating blood is a good maker of disease for infections caused by both *Mycobacterium bovis* and *Myocbacterium tuberculosis* (16–20). In all species of animal, mononucleocytes are the main targets of infection of mycobacterial pathogens in blood (21–23). When developing the phage-based assay, it was found that one of the critical steps was the removal of red blood cells (RBCs) from the sample as these can inhibit phage infection (16). Traditionally density gradient separation, such as the Ficoll buoyancy gradient method, has been widely used for the separation of monocytes and other white blood cells (24). However, this method is time consuming, requiring several centrifugation steps, and is sensitive to variation in both sample age and temperature. Recently, we have shown that differential sedimentation methods developed for the isolation of human white blood cells are compatible with the Actiphage assay (18). In addition we have shown that recovery of infected white blood cells from milk by centrifugation is also a suitable method to prepare samples prior to the phage assay (25).

In this study we wanted to investigate whether a differential lysis approach to remove the RBCs from a blood sample using Ammonium Chloride-Potassium (ACK) lysis buffer was compatible with the Actiphage assay. The Ammonium Chloride-Potassium (ACK) lysis method lyses erythrocytes as a result of osmotic stress caused by the uptake of ammonium chloride before cell debris is removed by washing in a buffer that is osmotically balanced for the white blood cells and has been shown to be effective for obtaining purified white blood cells from whole blood samples (26, 27). The performance of ACK lysis buffer method for the removal of RBCs was first compared to the traditional Ficoll method using cattle blood. The ACK method was then evaluated to see if it could be used to detect MAP in the blood of naturally infected, farmed deer.

## Materials and Methods

### Bacterial Strains and Media

MAP (strain K10; ATCC BAA-968) was used as the positive control for Actiphage assays. MAP was propagated in Middlebrook 7H9 broth (Difco, UK) supplemented with oleic albumin dextrose catalase (OADC) at a ratio of 1:10 and Mycobactin-J (2 μg ml^−1^; Synbiotic Corporation, France), grown with aeration at 37°C (28). The Actiphage (PBD Biotech Ltd., UK) phage reagent and Media Plus were prepared according to the manufacturer's instructions. Briefly Media Plus is prepared by supplementing the base media supplied in the kit with a sterile supplement at a 1 in 10 dilution rate. Media Plus is then used to reconstitute the freeze dried Actiphage reagent.

### Blood Samples

All clinical blood samples were handled under BS level 2 containment. For method optimisation experiments superfluous material from commercial bovine blood samples sent to PBD Biotech Ltd. for Actiphage testing was used. The clinical cervid blood samples originated from farmed deer in the East Midlands area of the UK. The deer were kept on outdoor pastures and in four different production groups. Blood was collected for diagnostic purposes by veterinarians in Heparin Vacutainer tubes (Beckton Dickinson, UK) and stored and transported at ambient temperatures (15–20°C) to ensure that the mycobacteria remained in an active growth phase required for productive D29 infection (28). The blood separation procedures were carried out within 12 h of collection. Samples for MAP blood ELISA assays were sent to Axiom Veterinary Laboratories (Devon, UK).

### Animal Groups for Testing

All the experiments in this study was performed using superfluous blood samples sent to PBD Biotech Ltd. for commercial testing and therefore were samples of convenience rather than taken from particular cohorts of animals of known infection status. Initial comparison of the Ficoll and ACK methods was carried out using superfluous blood from 43 cattle of unknown infection status, but with a known low herd prevalence of MAP infection.

All the deer samples were from females that were part of the commercial breeding stock on the farm which had experienced low levels of sporadic MAP infection in the past. Set 1 included 29 animals of a range of ages that were being screened before being sale. Blood samples from these animals were used to determine if the ACK method was compatible with deer blood, and to confirm that this method did not affect the performance of the Actiphage reagents. Set 2 included most of the mature breeding animals on the farm (ages 5–10 years) and were from different breeding groups and animals within these groups had shown signs on Johne's disease on previous occasions. These animals were used to determine whether the ACK/Actiphage method could detect MAP in the deer. Set 3 included only young animals that were being screened prior to the rut to identify which animals would be retained on the farm. This set was also used to determine whether MAP could be detected in the blood of these animals. A small number of animals from this group that gave a positive Actiphage results were included in a set of animals (set 4) that were screened using Actiphage 1 month later and allowed the reproducibility of the test results to be examined. Animals in set 5 were selected by the farm to be screened before sale using both MAP ELISA tests and Actiphage and included only animals that had previously been given a negative test result using Actiphage.

### Blood Preparation Methods

For separation of peripheral blood mononucleocytes (PBMCs) using Ficoll gradients (Ficoll paque Plus; GE Healthcare), a 10 ml Leucosep™ gradient tube (Greiner, Austria) was filled with 3 ml Ficoll density medium. 2.5 ml of Phosphate Buffered Saline (PBS) was gently mixed with a 2 ml sample of whole heparinised blood sample. The sample was then layered onto the top of the leucosep filter membrane. The sample was centrifuged (300 × g, 20 min, 19°C, swing out rotor with no deceleration). The buffy coat layer located above the filter membrane, was collected and mixed with 6 ml PBS before centrifuging (200 × g, 10 min, 19°C, swing out rotor with deceleration set at maximum. The pellet containing the blood mononucleocytes was resuspended in 1 ml of Actiphage Media Plus to induce lysis of the white blood cells and release the intracellular mycobacteria into the media. The samples were then either processed immediately (samples inoculated with laboratory cultured MAP) or incubated at room temperature for up to 12 h (clinical samples) prior to performing the Actiphage assay.

For purification of blood leukocytes (both mononucleocytes and granulocytes) using ACK buffer, a modification of the method described by Brown et al. (27) was used. Briefly, a sample (2 ml) of whole blood was added to 40 ml of ACK buffer (an aqueous solution of 150 mM Ammonium chloride, 10 mM Potassium bicarbonate, 0.1 mM EDTA, filter sterilised using 0.45 μm pore size). On addition of ACK the blood samples became more opaque, but this disappeared after gently agitating the sample for 5 min. The lysis process was determined to be complete when the sample became a dark red, transparent solution. The blood leukocytes were recovered by centrifugation (300 × g, 5 min at 20°C) and the supernatant discarded. The pellet was then washed twice to remove traces of erythrocyte debris using 5 ml of PBS with the intact white blood cells being recovered after each wash by centrifugation (300 × g, 5 min at 20°C). Finally the pellet was resuspended in 1 ml of Actiphage Media Plus and was stored at room temperature for upto 2 days before testing as described above.

### Actiphage Assay and qPCR

The Actiphage assay was carried out according to manufacturer's instruction. Briefly, after lysis of the white blood cells, any mycobacteria released into the Actiphage Media Plus were collected by centrifugation (13,000 × g, 3 min). The pellet was resuspended in 110 μl of rehydrated Actiphage reagent and then transferred into an Actiphage Rapid tube before incubating at 37°C for 3.5 h to allow phage absorption, infection and complete lysis of the mycobacterial cells. After the phage lysis step, the Actiphage Rapid tubes were centrifuged (13,000 × g, 3 min) and the lysate containing the released mycobacterial DNA was recovered from the collection tube. For the assay positive controls (Actiphage Media Plus inoculated with ~10^2^ MAP K10 cells) and negative controls (Media Plus and Actiphage reagent) were prepared according to the manufacturer's instructions.

DNA recovered from the Actiphage assay was concentrated using a Zymo™ clean and concentrator kit (Zymo, USA) and eluted in a total volume of 14 μl using 55°C water pre-warmed to 55°C. Detection of MAP DNA was carried out using the BactoReal kit for the detection of MAP (Ingenetix, Austria) which targets the IS900 element. qPCR reactions were performed according to the manufacturer's instructions using 5 μl of purified DNA per reaction. The positive control DNA sample provided with this kit contains ~ 10,000 target copies per reaction and the PCR Mastermix includes an Internal Positive control (IPC) labelled with VIC-TAMRA for detection of PCR inhibition. A test sample was determined positive if its Cq-value was 2 cycles lower than that of the Actiphage negative control sample (ΔCq ≥2). A sample producing a Cq-value between 1.5 and 2 units lower than the negative control was scored as weak positive (ΔCq =1.5–2).

### Statistical Analysis

Excel 2010 statistical add on package was used carry out a correlated *T*-Test when values for paired samples was compared. Significance was determined at *p* < 0.05. PCR efficiency was determined by plotting Ct-value against log_10_ (cell number) using Excel 2013, and determining the slope of the curve from the trend line and *R*^2^-values for each set of data points (*R*^2^ over 90% = acceptable). PCR efficiency was then calculated using the equation Efficiency (%) = [10^(−1/*slope*)^ – 1] × 100; acceptable range = 90–110%.

## Results

### Determining Compatibility of ACK Method With the Actiphage Assay

The Actiphage test is currently being performed by PBD Biotech Ltd. as a commercial blood test for detecting MAP in bovine blood samples and the kit instructions describe the isolation of white blood cells using a Ficoll density gradient method. To determine whether the ACK lysis method could be used as an alternative method to purify white blood cells prior to performing the Actiphage assay, superfluous blood from bovine samples set to PBD Biotech Ltd. were used. On the day of commercial testing, white blood cells were also prepared from blood of 43 cattle of unknown infection status using the ACK lysis method described by Brown et al. (27). Visible inspection showed that the ACK lysis method also produced purified white blood cells with low visible erythrocyte contamination which is key indicator for samples to be successfully tested using the Actiphage kit so that the results could be compared with the results reported for the commercial tests using the Ficoll method. Purified DNA was tested for the presence of MAP using a commercial qPCR kit (Bactoreal, Ingenetix) which includes an internal positive control (IPC) in the qPCR Mastermix to detect PCR inhibition. The signal from the IPC indicated that no PCR inhibition was detected when the DNA the samples prepared using either Ficoll or ACK were tested indicating that changing the extraction method did not result in a lower quality DNA sample (data not shown).

**Figure 1:**
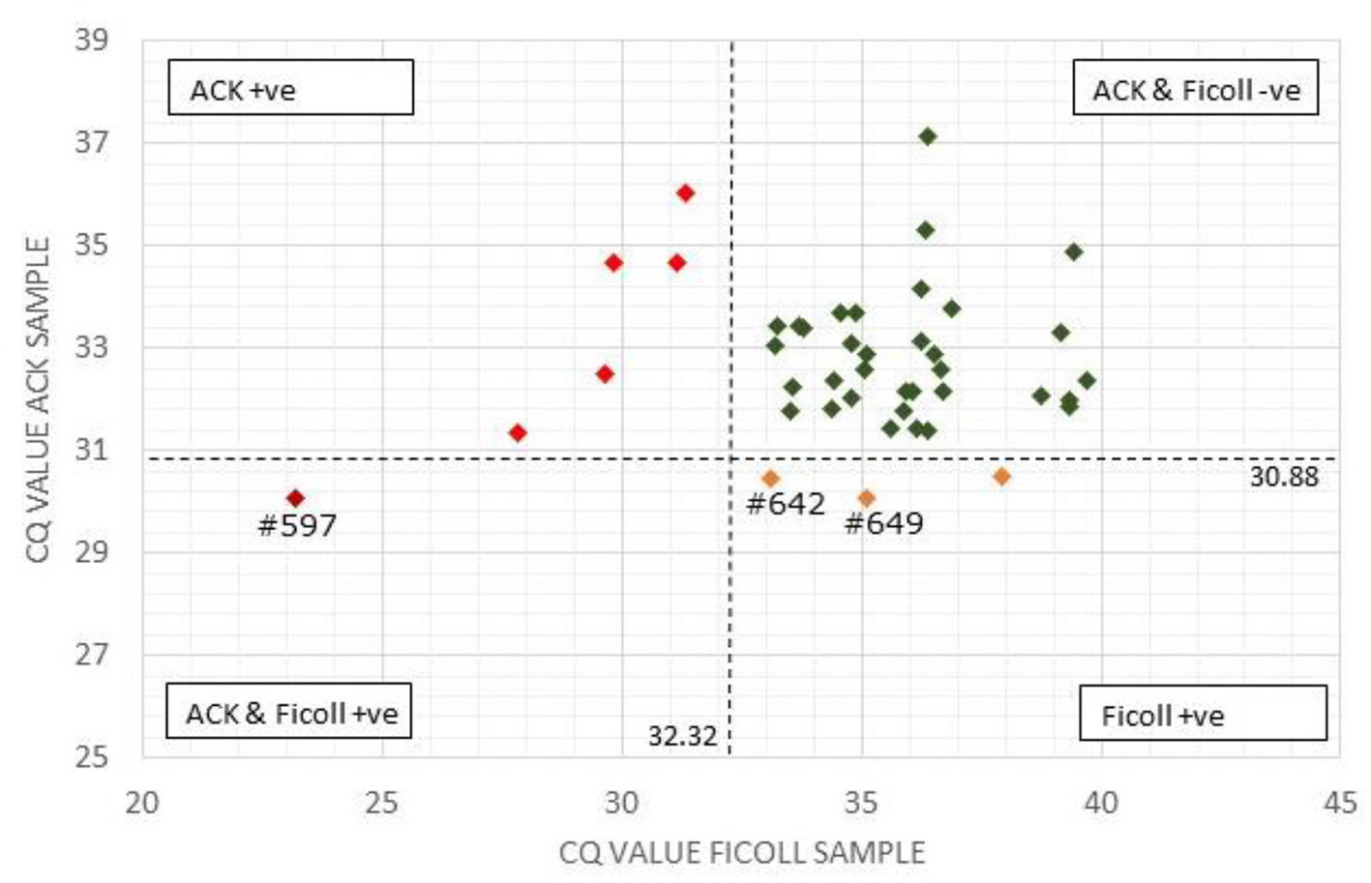
Comparison of Actiphage pPCR test results using Ficoll and ACK methods to purify white blood cells. The Cq-values for the paired blood samples are compared using a scatter plot. Vertical and horizontal dashed lines are set at the cut off values for the Ficoll and ACK samples, respectively. Samples falling within each quadrant are colour coded and cut off values are given next to each line. The position of individual samples (#) referred to in the text are indicated.

The IS900 target DNA positive control sample provided in the kit that contained ~10,000 copies of the MAP-specific target sequence gave a Cq-value of 20.54 (expected range 19–22). For the Actiphage assay, a positive control containing ~10^2^ freshly grown MAP K10 cells is used, and in this experiment produced a Cq-value of 21.13. Based on the Cq-value of the positive control the expected range for the MAP positive control would be 21–22 as the K10 strain contains 17 copies of the IS900 element (i.e., 10^2^ cells contain 1,700 copies of the IS900 target sequence). For the Actiphage test, a negative control sample is also prepared using the media supplied in the kit and the Actiphage reagent alone but still contain high levels of phage DNA and residual DNA from the *Mycobacterium smegmatis* strain used to propagate the Actiphage reagent which may result in PCR bridging events. In this case the negative control samples give Cq-values in the range of 30–32 and therefore these values are used to establish a base line for identifying positive test results (see Figure 1).

A comparison of the qPCR analysis of the 43 bovine samples prepared using the two different methods is shown in Figure 1. Using the standard Ficoll isolation method, MAP DNA was detected in 4 of the clinical bovine blood samples, two with a clear positive result (ΔCq = ≥2; #597, #649) and two gave a weak positive result (ΔCq = 1.5–2) indicating that very low levels of Map (~5 cells) had been detected. In comparison, using the ACK lysis method, 6 positive results and no weak positive results were obtained. Comparing the pattern of results, the sample with the highest ΔCq-value using the ACK group matched one of the two positive results for the Ficoll samples (Figure 1; #597). Animal #642 gave a weak positive result with the Ficoll method and had a ΔCq of 1.2 for the ACK method, indicating that it was just below the threshold for a weak positive score. The two other positive results from the Ficoll set gave negative test results using ACK. When low levels of MAP are being detected in samples, some stochastic variation is number of cells per sample is to be expected [see (25)].

For PCR efficiency determination, 10-fold dilutions of a MAP positive control culture with a starting titre of 1.2 × 10^4^ cfu ml^−1^ were used to inoculate white blood cells extracted using the two different methods with cell numbers from ~10–10^4^ cells. For the Ficoll method the PCR efficiency value was 89.6% (*R*^2^ = 0.996), with an intercept Ct-value of 30.85, whereas for the ACK method the PCR efficiency value as 98.2% (*R*^2^ = 0.994), with an intercept Ct-value of 31.24 indicating that sensitivity achieved using the two sample preparation methods was very similar (Supplementary Figure 1). Overall from these results it was concluded that the ACK method was compatible with the Actiphage reagents as there was no evidence of inhibition and positive test results were achieved.

### Comparison of Blood Processing Methods for Clinical Cervid Blood Samples

Ficoll can be used to isolate white blood cells from many species of animal, including cervids, and ACK has been shown to be able to remove contaminating erythrocytes from cervid buffy coat preparations (29). However, deer blood is known to exhibit unusual properties due to sickling which can be associated with increased fragility of erythrocytes (30). Therefore, to determine whether the ACK lysis method was an appropriate method for bulk isolation of white blood cells from whole blood, surplus material from 29 deer blood samples (set 1) provided for commercial Actiphage testing were obtained. White blood cells were recovered from 2 ml samples using either the recommended Ficoll method or the ACK lysis method. Although buffy coat was recovered from deer blood samples using Ficoll, the position of the buffy coat layer was inconsistent, and often formed a layer underneath the porous frit of the Leucosep tubes making extraction more difficult. In contrast a pellet of purified blood leukocytes was formed using the ACK method that on visible inspection was cream in colour indicating very little contamination with erythrocytes.

After the Actiphage assay had been performed, no PCR inhibition was detected by the IPC for either set of DNA samples, again confirming that the ACK method was compatible with the Actiphage assay reagents. Similarly the Actiphage assay positive control (~10^2^ MAP cells) and negative control (Actiphage reagents) samples gave Cq-values in the expected range (Table 1) but for this set of animals no clear positive test results were obtained, although tests were scored as weak positive for 4 animals (Table 1). The Cq-values for the paired samples was compared (Table 1) and were not found to be statistically different (*p* = 0.16), with an average ACK:Ficoll Cq of 0.99 with only 1 sample (#21) significantly deviating from this value. These results indicated that using the ACK method of white blood cell preparation did not affect the assay performance.

**Table 1:** Comparison of qPCR result for deer blood samples prepared using Ficoll and ACK methods.

**Sample**	**Blood preparation method**				**Ratio ACK:Ficoll Cq^**c**^**
	**ACK Cq**	**ΔCq^**b**^**	**Ficoll Cq**	**ΔCq^**b**^**	
**Act**. **+ve**^**a**^	20.83	n/a	22.24	n/a	0.94
**Act. –ve** ^**a**^	30.29	n/a	32.36	n/a	0.94
1	33.45	−3.2	40.72	−8.4	0.82
2	36.09	−5.8	35.49	−3.1	1.02
3	33.32	−3.0	32.51	−0.1	1.02
5	33.94	−3.7	33.00	−0.6	1.03
6	32.38	−2.1	32.33	0.0	1.00
7	34.40	−4.1	38.02	−5.7	0.90
8	33.57	−3.3	34.80	−2.4	0.96
9	28.38	1.9	33.76	−1.4	0.84
10	32.40	−2.1	34.00	−1.6	0.95
11	33.03	−2.7	34.35	−2.0	0.96
12	33.61	−3.3	33.86	−1.5	0.99
13	32.86	−2.6	31.86	0.5	1.03
14	33.63	−3.3	33.80	−1.4	0.99
15	32.79	−2.5	30.48	1.9	1.08
16	31.85	−1.6	33.44	−1.1	0.95
17	33.10	−2.8	35.57	−3.2	0.93
19	31.94	−1.7	33.84	−1.5	0.94
20	31.32	−1.0	31.96	0.4	0.98
21	43.93	−13.6	31.40	1.0	**1.40**
22	36.38	−6.1	35.09	−2.7	1.04
23	31.95	−1.7	36.73	−4.4	0.87
24	31.40	−1.1	35.03	−2.7	0.90
25	33.29	−3.0	34.40	−2.0	0.97
26	33.10	−2.8	33.20	−0.8	1.00
27	32.20	−1.9	30.82	1.5	1.04
29	31.95	−1.7	30.62	1.7	1.04

a *Actiphage positive control = Media Plus inoculated with ~10^2^ MAP K10 cells; Actiphage negative control = Media Plus and Actiphage reagents alone*.

b *ΔCq, difference in Cq sample compared to the Actiphage negative control; n/a, not applicable; Positive test result = ΔCq = ≥2; A weak positive test result = ΔCq 1.5-2 (weak positive results shaded in grey)*.

c *Ratio of Cq-values = Cq[ACK]/Cq[Ficoll] for each paired sample. Average value = 0.99. The one sample where this ratio was significantly larger highlighted in bold*.

### Detecting MAP in Deer Blood

**Figure 2:**
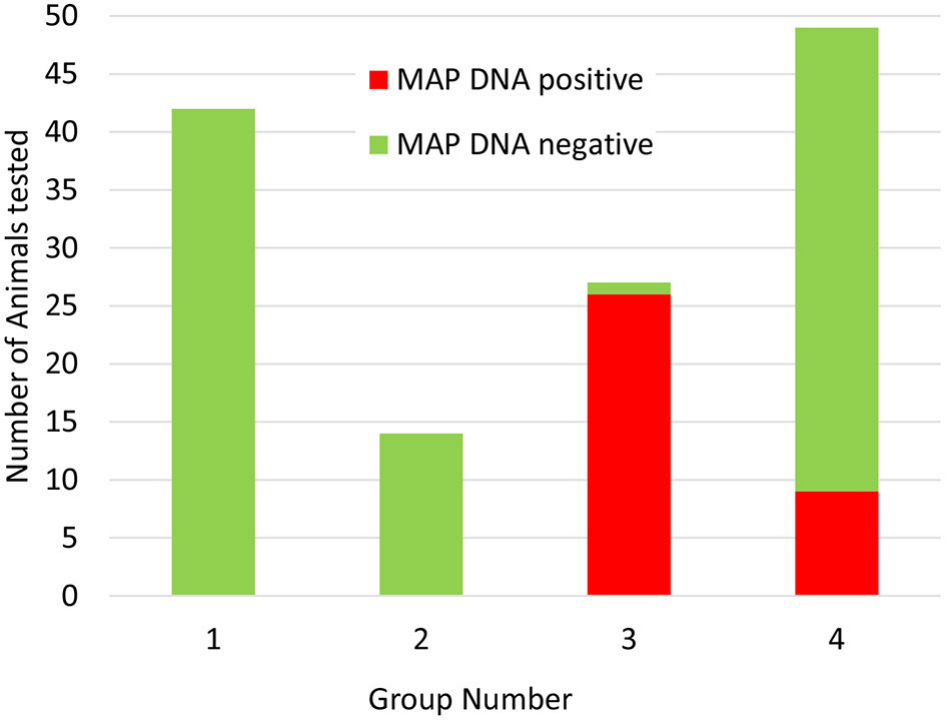
Distribution of Actiphage MAP test results in commercial deer herd production groups. Blood samples were taken from 132 female deer prior to the rutting season. The samples were supplied to the lab blinded to production group. After the test results were available, the samples were unblended and sorted by production group. Green bars indicate negative MAP test results; red bars indicate positive MAP test results.

Having established that the ACK method could be used to purify leukocytes from whole deer blood, the method was used to screen blood samples from a set of 132 female red deer immediately prior to the autumn rutting season (set 2). Again, positive and negative control samples gave the expected results and no qPCR inhibition was detected. In this set of animals MAP was detected in the blood samples of 35 animals (27%). Approximately 2/3 of the positive samples (21/31) produced strong positive results (ΔCq > 5), and the remaining 10 samples were scored as weak positive (ΔCq 1.5–2). Interestingly, when the animals were assigned to their production groups, it was found that all but nine of the animals that gave a positive test results belonged to one production group, and the rest were all found to belong to a second production group (see Figure 2 and Supplementary Table 1).

The following year, a set of 298 yearling animals from the same farm were tested using the ACK blood preparation method but as the farm only chose to test young animals on this occasion, this group did not include any of the animals tested the previous year (set 3). In this set of animals, 16% gave a positive test result (Supplementary Table 2). The following month a small set of 11 animals from this group that gave a positive test result were included in the blood samples set for screening (set 4; Table 2). At the second time of sampling all these animals again gave a positive test result, although 3 only gave a weak positive results (ΔCq 1.5–2).

**Table 2:** Comparison of Actiphage-test results after 1 month.

**Sample**	**Test date: 27/08/20**			**Test date: 22/09/20**		
	**Test result^**b**^**	**Cq-value**	**ΔCq^**c**^**	**Test result^**b**^**	**Cq-value**	**ΔCq^**c**^**
**Act**. **+ve**^**a**^	+ve	25.72	n/a	+ve	18.68	n/a
**Act. –ve** ^**a**^	-ve	30.43	n/a	–ve	32.44	n/a
1	+ve	21.44	9.0	+ve	29.75	2.7
2	+ve	22.34	8.1	+ve (w)	30.97	1.5
3	+ve	22.97	7.5	+ve	29.78	2.7
4	+ve	23.15	7.3	+ve (w)	30.59	1.9
5	+ve	24.98	5.5	+ve	29.25	3.2
6	+ve	26.02	4.4	+ve (w)	30.49	1.9
7	+ve	26.22	4.2	+ve	29.34	3.1
8	+ve	27.43	3.0	+ve	29.95	2.5
9	+ve	27.96	2.5	+ve	29.61	2.8
10	+ve	28.05	2.4	+ve	30.21	2.2
11	+ve	28.09	2.3	+ve	30.22	2.2

*All animals tested were female, and were born in the same year (2019)*.

a *Actiphage positive control = Media Plus inoculated with ~10^2^ MAP K10 cells; Actiphage negative control = Media Plus and Actiphage reagents alone*.

b *Test result; Positive = ΔCq = ≥2; Positive weak (w) = ΔCq 1.5-2*.

c *ΔCq; difference in Cq sample compared to the Actiphage negative control; n/a, not applicable*.

A set of 38 animals were also chosen by the farm for commercial testing prior to sale that had given a negative test result the previous year and provided an opportunity to investigate the reproducibility of negative test results (set 5). On this occasion, blood samples were taken for both commercial MAP blood ELISA and Actiphage testing using the ACK method. In this case all blood samples from these animals produced a negative Actiphage test result and the ELISA test results were also all negative (data not shown).

## Discussion

We have previously shown that phage-based assays can be used to detect MAP in blood mononucleocytes of infected cattle (16) and more recently have described a new, more rapid and sensitive phage-based test that can be used to detect infection at any age [Actiphage; (17)]. In these previous studies Ficoll gradients were used to recover blood mononucleocytes, however this method has its disadvantages in that it is laborious and requires costly reagents. In this study we have demonstrated that the ACK differentiation lysis method, developed for the rapid recovery of stem cells, is compatible with the Actiphage assay. Unlike INF-γ assays, the physiological state of the white blood cells is not important for phage-based detection assays. Rather it is the physiological state of the MAP cells that is critical, and the leukocytes are lysed during the first steps of the assay to release any intracellular MAP into a growth medium to keep them in a metabolically active state required for phage infection (28). Indeed the additional osmotic stress that the leukocytes experience during the ACK procedure may make them more susceptible to lysis which would also facilitate phage-based detection.

When preparing samples from deer blood, it was noted that there was less detectable erythrocyte contamination when using the ACK method. Contamination of the Ficoll purified buffy coats may have been due to the fact that the buffy coat layer was formed very close to the surface of the erythrocyte layer, some of which may have been picked up while pipetting off the delicate buffy coat layer or may be due to the higher fragility of the erythrocytes causing premature lysis (30). Hence for cervid blood samples, the ACK method had another advantage over standard Ficoll gradients.

In this study, the ACK method was used to screen animals from a commercial herd of farmed deer. The farm had had sporadic problems with JD after some animals had been purchased, but no cases of clinical disease had been detected recently. Initially a set of female animals were tested, with blood samples provided simply in the order the animals entered the testing pen. In this set of results there was an unusual pattern, with a cluster of positive results occurring in samples collected at the end of the process. This pattern of results was explained when the test results were compared with information provided about the production groups, when it became evident that there was one main group (group 3) which had the highest level of MAP infection and a lower level of infection detected in group 4, whereas groups 1 and 2 did not give any positive test results. Since these groups are geographically separated on the farm, this information is now being integrated into the farm management plan to ensure that infected animals are not moved to the uninfected groups, and animals known to be infected are being prioritised for culling when numbers need to be reduced.

When the farm testing program allowed, it was also possible to carry out some small scale test reproducibility evaluation work, and it was found that when repeat tests were performed within 1 month that the results gained were consistent. The variation in the levels of DNA detected in the same animal (Table 2) is to be expected as there will be stochastic variation in the number of MAP cells detected when only very low number of infected macrophage are present in the blood and only small (2 ml) blood samples are being tested [see (25)]. Our previous studies of MAP in bovine blood have detected only 5–50 MAP cells per sample using phage-based assays (16, 31) and therefore only a very small percentage of the PBMCs in a sample (~10^6^) are likely to be infected. If larger samples could be tested, this would reduce the stochastic variation, but currently the viscosity of the sample which contains large amounts of bovine DNA derived from the lysed macrophages prevents larger volumes of blood processed using the Actiphage kit. Similarly when a set of animals that had previously given a negative Actiphage test result were tested again using both ELISA and Actiphage, both tests gave negative results. Since a commercial qPCR kit was used to detect the MAP DNA, and the specificity of the detection event comes from the qPCR step, this provides confidence that these are true positive results. However, it was also clear that this non-optimised qPCR assay resulted in high background noise levels. The Bactoreal kit is optimised for the detection of MAP DNA in faecal samples and PCR bridging can lead to positive Cq-values when the sample DNA contains high concentrations of off targets and primer concentrations have not been optimised (32–34). If the PCR reagents are optimised, this may allow lower levels of cells to be detected with confidence. Further studies are required to fully validate the Actiphage method for detecting MAP infections in deer, including comparison with other standard diagnostic test methods, but these results provide first evidence that Actiphage is specific and in combination with the ACK lysis method can be used to detect MAP infection in farmed deer.

The inherent specificity of bacteriophage D29 towards its Mycobacterial host cells, coupled with the high specificity and sensitivity of PCR-based detection methods has led to the development of the new, more sensitive Actiphage assay. The replacement of Ficoll gradients further simplifies the method and the chemicals required for the production of ACK lysis buffer (Ammonium chloride, Potassium bicarbonate and EDTA) are widely available and inexpensive. This proof of principle study demonstrates that Actiphage is a new tool that can directly detect the presence of MAP in cervid blood samples and clearly has potential for the control of Johne's disease.

## Data Availability Statement

The original contributions presented in the study are included in the article/Supplementary Material, further inquiries can be directed to the corresponding author.

## Ethics Statement

Ethical review and approval was not required for the animal study because Superfluous blood samples from commercial testing program provided. Written informed consent was obtained from the owners for the participation of their animals in this study.

## Author Contributions

AK developed and evaluated the ACK method for use with deer blood, and contributed to writing the manuscript. AP and CE generated results for commercial samples using the Ficoll method. TP and BS contributed to the design, supervision of experiments, writing, and reviewing the manuscript. CR contributed to design of experiments, data analysis, and writing the manuscript. All authors contributed to the article and approved the submitted version.

## Conflict of Interest

CR and BS are founder members and shareholders of PBD Biotech Ltd., and AP is an employee of the company. AP and CE are UoN students sponsored by the company, but they can declare that the research was conducted in the absence of any commercial or financial relationships that could be construed as a potential conflict of interest. TP is an employee of UoN and has no commercial or financial relationship with the company. The remaining author declares that the research was conducted in the absence of any commercial or financial relationships that could be construed as a potential conflict of interest.

## Publisher's Note

All claims expressed in this article are solely those of the authors and do not necessarily represent those of their affiliated organizations, or those of the publisher, the editors and the reviewers. Any product that may be evaluated in this article, or claim that may be made by its manufacturer, is not guaranteed or endorsed by the publisher.
