# Supplementary material for: Development of a Method to Detect Mycobacterium paratuberculosis in the Blood of Farmed Deer Using Actiphage® Rapid
Source: Front Vet Sci. 2021 Jul 29;8:665697. doi: 10.3389/fvets.2021.665697 (PMC8358306; doi:10.3389/fvets.2021.665697)
Supplement: Supplementary file 1 [file Data_Sheet_1.PDF]

Table S1: qPCR data for Deer Set 2 (sampled 2019)

SAMPLE NO	Cq	ΔCq[d]	TEST RESULTS INTERPRETATION[e]	PRODUCTION GROUP NO.[f]
**1**	45	-12.14	negative	1
**2**	35.04	-2.18	negative	1
**3**	35.85	-2.99	negative	1
**4**	45	-12.14	negative	1
**5**	35.6	-2.74	negative	1
**6**	33.29	-0.43	negative	1
**7**	35.4	-2.54	negative	1
**8**	34.92	-2.06	negative	1
**9**	35.83	-2.97	negative	1
**10**	34.2	-1.34	negative	1
**11**	35.54	-2.68	negative	1
**12**	36.04	-3.18	negative	1
**13**	45	-12.14	negative	1
**14**	36.51	-3.65	negative	1
**15**	35.5	-2.64	negative	1
**16**	45	-12.14	negative	1
**17**	45	-12.14	negative	1
**18**	34.65	-1.79	negative	1
**19**	34.55	-1.69	negative	1
**20**	34.89	-2.03	negative	1
**21**	37.96	-5.1	negative	1
**22**	35.74	-2.88	negative	1
**23**	35.48	-2.62	negative	1
**24**	36.03	-3.17	negative	1
**25**	36.65	-3.79	negative	1
**26**	32.76	0.1	negative	1
**27**	32.1	0.76	negative	1
**28**	33.54	-0.68	negative	1
**29**	34.14	-1.28	negative	1
**30**	34.36	-1.5	negative	1
**31**	33.77	-0.91	negative	1
**32**	35.09	-2.23	negative	1
**33**	34.05	-1.19	negative	1
**34**	33.12	0.16	negative	1
**35**	35.95	-3.09	negative	1
**36**	45	-12.14	negative	1
**37**	35.06	-2.2	negative	1
**38**	36.26	-3.4	negative	1
**39**	45	-12.14	negative	1
**40**	35.67	-2.81	negative	1
**41**	33.45	-0.59	negative	1
**42**	33.91	-1.05	negative	1
**43**	37.43	-4.57	negative	4

**44**	41.49	-8.63	negative	4
**45**	33.64	-0.78	negative	4
**46**	36.09	-3.23	negative	4
**47**	45	-12.14	negative	4
**48**	35.05	-2.19	negative	4
**49**	35.35	-2.49	negative	4
**50**	31.27	1.59	weak positive	4
**51**	35.07	-2.21	negative	4
**52**	33.27	-0.41	negative	4
**53**	34.12	-1.26	negative	4
**54**	34.3	-1.44	negative	4
**55**	33.23	-0.37	negative	4
**56**	33	-0.14	negative	4
**57**	34.33	-1.47	negative	4
**58**	34.29	-1.43	negative	4
**59**	33.45	-0.59	negative	4
**60**	34.28	-1.42	negative	4
**61**	33.57	-0.71	negative	4
**62**	35.17	-2.31	negative	4
**63**	33.63	-0.77	negative	4
**64**	33.37	-0.51	negative	4
**65**	34.58	-1.72	negative	4
**66**	34.42	-1.56	negative	4
**67**	33.74	0.96	negative	4
**68**	33.75	0.95	negative	4
**69**	33.85	0.85	negative	4
**70**	33.85	0.85	negative	4
**71**	34.95	-0.25	negative	4
**72**	35.45	-0.75	negative	4
**73**	33.33	1.37	negative	4
**74**	34.5	0.2	negative	4
**75**	33.57	1.13	negative	4
**76**	33.11	1.59	weak positive	4
**77**	33.56	1.14	negative	4
**78**	32.12	2.58	positive	4
**79**	35.1	-0.4	negative	4
**80**	34.79	-0.09	negative	4
**81**	45	-10.3	negative	4
**82**	34.67	0.03	negative	4
**83**	31.22	3.48	positive	4
**84**	35.61	-0.91	negative	4
**85**	33.55	1.15	negative	4
**86**	33.41	1.29	negative	4
**87**	34.08	0.62	negative	4
**88**	32.44	2.26	positive	4
**89**	33.58	1.12	negative	4

**90**	33.8	0.9	negative	4
**91**	33.32	1.38	negative	4
**92**	32.51	2.19	positive	3
**93**	32.93	1.77	weak positive	3
**94**	32.59	2.11	positive	3
**95**	33.18	1.52	weak positive	3
**96**	33.15	1.55	weak positive	3
**97**	32.27	2.43	positive	3
**98**	31.23	3.47	positive	3
**99**	35	-0.3	negative	3
**100**	31.89	2.81	positive	3
**101**	34.68	0.02	negative	2
**102**	30.69	4.01	positive	3
**103**	32.12	2.58	positive	3
**104**	31.79	2.91	positive	3
**105**	32.08	2.62	positive	3
**106**	33.55	1.15	negative	2
**107**	32.7	2.00	positive	3
**108**	32.51	2.19	positive	3
**109**	31.68	3.02	positive	3
**110**	32.42	2.28	positive	3
**111**	33.4	1.3	negative	2
**112**	32.62	2.08	positive	3
**113**	34.25	0.45	negative	2
**114**	33.67	1.03	negative	2
**115**	32.24	2.46	positive	3
**116**	34.04	0.66	negative	2
**117**	33.04	1.66	weak positive	3
**118**	33.44	1.26	negative	2
**119**	33.76	0.94	negative	2
**120**	32.47	2.23	positive	3
**121**	32.62	2.08	positive	3
**122**	34.25	0.45	negative	2
**123**	32.68	2.02	weak positive	3
**124**	33.06	1.64	weak positive	3
**125**	33.49	1.21	negative	2
**126**	33.08	1.62	weak positive	3
**127**	33.31	1.39	negative	2
**128**	34.92	-0.22	negative	2
**129**	33.2	1.5	weak positive	3
**130**	33.19	1.51	weak positive	3
**131**	33.43	1.27	negative	2
**132**	36.36	-1.66	negative	2
**RUN 1 CONTROLS**				
**WATER**	45		negative	

**MEDIA + PHAGE**[a]	32.86	negative
**MAP CELLS**[b]	20.91	positive
**INGENETIX +VE**[c]	23.65	positive
**RUN 2 CONTROLS**		
**WATER**	45	negative
**MEDIA + PHAGE**[a]	34.7	negative
**MAP CELLS**[b]	17.71	positive
**INGENETIX +VE**[c]	23.38	positive

[a] Actiphage negative control = Media Plus and Actiphage reagents alone.

[b]MAP CELLS = Media Plus inoculated with approximately 10^2 MAP K10 cells

[c]DNA positive control provided with Ingenetix Bactoreal kit

[d] ΔCq; difference in Cq sample compared to the Actiphage negative control. n/a = not applicable

[e] Test interpretation; Positive = ΔCq=≥2; weak positive weak = ΔCq 1.5-2; ΔCq <2 = negative.

[f]Data is ordered by sample number as provided to the laboratory. Production group number indicates which of the production groups the animals were taken from. This information was used to sort the data to generate Figure 2.

Figure S1. Efficient plot comparing effect of ACK and Ficoll methods on PCR

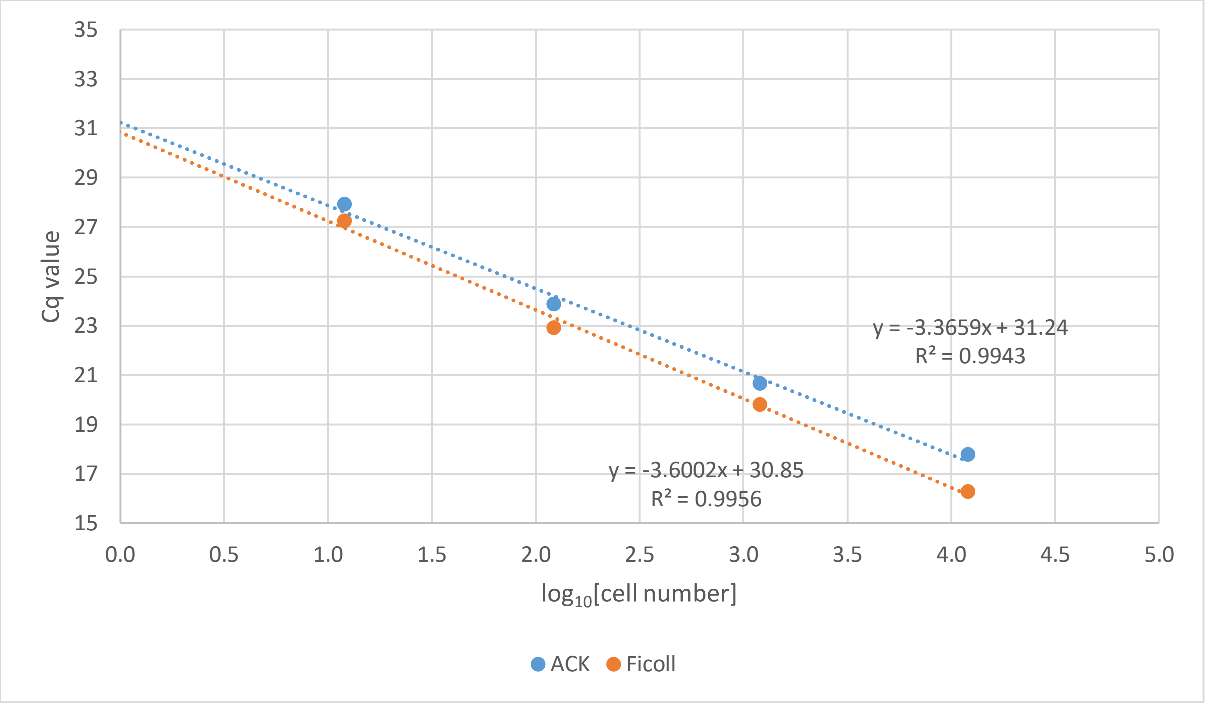

	% eff	intercept
Ficoll	89.6	31.24
ACK	98.2	31.78

PCR efficiency was determined by plotting Ct value against log_{10}[cell number] and determining the slope of the curve from the trend line and R^2 values for each set of data points (R^2 over 90% = acceptable). PCR efficiency was then calculated using the equation Efficiency (%) = $(10^{(-1/slope)}$-1)x100; acceptable range = 90-110%.
