# Supplementary material for: Development of a Method to Detect Mycobacterium paratuberculosis in the Blood of Farmed Deer Using Actiphage® Rapid
Source: Front Vet Sci. 2021 Jul 29;8:665697. doi: 10.3389/fvets.2021.665697 (PMC8358306; doi:10.3389/fvets.2021.665697)
Supplement: Supplementary file 2 [file Data_Sheet_2.PDF]

week 1

First Run			Green MAP	ΔCq	No. Positive
Position	Sample name	Sample type	Cq Value		
1	Lab ID: 1	TestSample	39.65	-6.60	
2	Lab ID: 2	TestSample	40.15	-7.10	
4	Lab ID: 4	TestSample	40.52	-7.47	
6	Lab ID: 6	TestSample	44.71	-11.66	
13	Lab ID: 13	TestSample	40.29	-7.24	
14	Lab ID: 14	TestSample	39.33	-6.28	
15	Lab ID: 15	TestSample	31.06	2.0	1
16	Lab ID: 16	TestSample	39.49	-6.44	
19	Lab ID: 19	TestSample	40.54	-7.49	
24	Lab ID: 24	TestSample	55.00	-21.95	
25	Lab ID: 25	TestSample	40.82	-7.77	
27	Lab ID: 27	TestSample	31.51	1.54	2
28	Lab ID: 28	TestSample	31.52	1.53	3
29	Lab ID: 29	TestSample	40.94	-7.89	
30	Lab ID: 30	TestSample	41.37	-8.32	
31	Lab ID: 31	TestSample	39.98	-6.93	
32	Lab ID: 32	TestSample	39.96	-6.91	
33	Lab ID: 33	TestSample	40.45	-7.40	
40	Lab ID: 40	TestSample	39.89	-6.84	
41	Lab ID: 41	TestSample	42.41	-9.36	
42	Lab ID: 42	TestSample	41.12	-8.07	
43	Lab ID: 43	TestSample	41.56	-8.51	
45	Lab ID: 45	TestSample	40.00	-6.95	
46	Lab ID: 46	TestSample	41.75	-8.70	
49	Lab ID: 49	TestSample	32.13	0.92	
50	Lab ID: 50	TestSample	32.14	0.91	
51	Lab ID: 51	TestSample	32.38	0.67	
52	Lab ID: 52	TestSample	30.75	2.30	4
53	Lab ID: 53	TestSample	32.27	0.78	
54	Lab ID: 54	TestSample	32.41	0.64	
55	Lab ID: 55	TestSample	30.86	2.19	5
56	Lab ID: 56	TestSample	32.11	0.94	
57	Lab ID: 57	TestSample	32.56	0.49	
58	Lab ID: 58	TestSample	32.26	0.79	
59	Lab ID: 59	TestSample	32.13	0.92	
60	Lab ID: 60	TestSample	32.27	0.78	
61	Lab ID: 61	TestSample	32.74	0.31	
62	Lab ID: 62	TestSample	32.11	0.94	
63	Lab ID: 63	TestSample	32.13	0.92	
64	Lab ID: 64	TestSample	32.05	1.00	
65	Lab ID: 65	TestSample	32.59	0.46	
66	Lab ID: 66	TestSample	32.78	0.27	

67	MP/Actiphage	NegativeControl	33.05	n/a
69	MAP -2	PositiveControl	20.92	n/a
70	Kit Control EPC	PositiveControl	21.93	n/a

No.	42

week 1

Second Run			Green MAP	ΔCq	
Position	Sample name	Sample type	Cq Value		
1	Lab ID: 3	TestSample	33.40	-1.64	
2	Lab ID: 5	TestSample	32.58	-0.82	
3	Lab ID: 7	TestSample	33.76	-2.00	
4	Lab ID: 8	TestSample	28.55	3.21	6
5	Lab ID: 9	TestSample	33.84	-2.08	
6	Lab ID: 10	TestSample	33.25	-1.49	
7	Lab ID: 11	TestSample	33.99	-2.23	
8	Lab ID: 12	TestSample	34.14	-2.38	
9	Lab ID: 17	TestSample	31.78	-0.02	
14	Lab ID: 23	TestSample	35.75	-3.99	
15	Lab ID: 26	TestSample	29.25	2.51	7
16	Lab ID: 35	TestSample	30.64	1.12	
17	Lab ID: 36	TestSample	33.76	-2.00	
18	Lab ID: 44	TestSample	27.60	4.16	8
19	Lab ID: 47	TestSample	29.31	2.45	9
20	Lab ID: 48	TestSample	32.33	-0.57	
21	Lab ID: 49	TestSample	24.75	7.01	10
22	Lab ID: 50	TestSample	25.37	6.39	11
23	Lab ID: 67	TestSample	32.32	-0.56	
24	Lab ID: 68	TestSample	35.11	-3.35	
25	Lab ID: 69	TestSample	34.08	-2.32	
26	Lab ID: 70	TestSample	34.79	-3.03	
27	Lab ID: 71	TestSample	34.52	-2.76	
28	Lab ID: 72	TestSample	34.37	-2.61	
29	Lab ID: 73	TestSample	35.44	-3.68	
30	Lab ID: 74	TestSample	34.48	-2.72	
31	Lab ID: 75	TestSample	34.49	-2.73	
32	MP/Actiphage	NegativeControl	33.76	n/a	
33	Kit EPC positive	PositiveControl	20.52	n/a	

No.	27
Total	69

week 2

First Run			Green MAP	ΔCq	
Position	Sample name	Sample type	Cq Value		
1	76	TestSample	31.30	-1.51	
2	77	TestSample	30.01	-0.22	
3	78	TestSample	30.26	-0.47	
4	79	TestSample	30.10	-0.31	
5	80	TestSample	27.70	2.09	12
6	81	TestSample	30.65	-0.86	
7	82	TestSample	30.79	-1.00	

8	83	TestSample	30.02	-0.23	
9	84	TestSample	30.51	-0.72	
10	85	TestSample	31.00	-1.21	
11	86	TestSample	30.59	-0.80	
12	87	TestSample	30.70	-0.91	
13	88	TestSample	30.06	-0.27	
14	89	TestSample	28.57	1.22	
15	90	TestSample	28.92	0.87	
16	91	TestSample	27.01	2.78	13
17	92	TestSample	29.07	0.72	
18	93	TestSample	30.93	-1.14	
23	98	TestSample	25.65	4.14	14
24	99	TestSample	29.56	0.23	
25	100	TestSample	28.74	1.05	
26	101	TestSample	25.28	4.51	15
27	102	TestSample	28.12	1.67	16
28	103	TestSample	29.46	0.33	
29	104	TestSample	28.89	0.90	
30	105	TestSample	29.05	0.74	
31	106	TestSample	29.77	0.02	
32	107	TestSample	28.46	1.33	
33	108	TestSample	29.44	0.35	
34	109	TestSample	30.08	-0.29	
35	110	TestSample	28.99	0.80	
36	111	TestSample	29.11	0.68	
37	112	TestSample	29.52	0.27	
38	113	TestSample	28.75	1.04	
39	114	TestSample	29.25	0.54	
40	115	TestSample	29.67	0.12	
41	116	TestSample	29.81	-0.02	
42	117	TestSample	29.53	0.26	
43	118	TestSample	29.93	-0.14	
44	119	TestSample	29.23	0.56	
45	120	TestSample	29.68	0.11	
46	121	TestSample	29.66	0.13	
47	122	TestSample	29.81	-0.02	
49	124	TestSample	26.78	3.01	17
51	126	TestSample	27.91	1.88	18
55	130	TestSample	26.86	2.93	19
56	131	TestSample	20.00	9.79	29
57	132	TestSample	29.15	0.64	
58	133	TestSample	21.66	8.13	21
59	134	TestSample	29.01	0.78	
62	137	TestSample	29.11	0.68	
63	138	TestSample	23.89	5.90	22
65	140	TestSample	21.14	8.65	23
66	MP/Actiphage	NegativeControl	29.29	n/a	
69	MAP -2	PositiveControl	25.30	n/a	
70	Kit Control EPC	PositiveControl	16.93	n/a	

No.	53
Total	122
week 2	

Second Run			Green MAP	ΔCq	
Position	Sample name	Sample type	Cq Value		
12	127	TestSample	30.47	-0.79	
13	128	TestSample	26.22	3.46	12
14	129	TestSample	30.48	-0.80	
15	135	TestSample	26.43	3.25	14
16	136	TestSample	27.90	1.78	15
17	139	TestSample	29.80	-0.12	
18	141	TestSample	23.14	6.54	13
19	142	TestSample	22.97	6.71	14
20	143	TestSample	30.22	-0.54	
21	144	TestSample	24.18	5.50	15
22	145	TestSample	27.90	1.78	16
23	146	TestSample	26.44	3.24	17
24	147	TestSample	30.28	-0.60	
25	148	TestSample	30.18	-0.50	
26	MP/Actiphage	NegativeControl	29.68	n/a	
27	MAP -2	PositiveControl	25.72	n/a	

No.	18
Total	140
week 3	

First Run			Green MAP	ΔCq	
Position	Sample name	Sample type	Cq Value		
1	Set1: 25	TestSample	29.21	1.51	24
2	26	TestSample	29.75	0.97	
3	27	TestSample	29.97	0.75	
4	28	TestSample	28.36	2.36	25
5	29	TestSample	29.77	0.95	
6	30	TestSample	29.50	1.22	
7	31	TestSample	28.50	2.22	26
8	32	TestSample	31.03	-0.31	
9	33	TestSample	29.66	1.06	
10	34	TestSample	30.42	0.30	
11	35	TestSample	30.56	0.16	
12	36	TestSample	31.05	-0.33	
13	37	TestSample	29.57	1.15	
14	38	TestSample	28.30	2.42	27
15	39	TestSample	29.41	1.31	
16	40	TestSample	29.57	1.15	
17	41	TestSample	30.23	0.49	
18	42	TestSample	30.44	0.28	
23	43	TestSample	28.24	2.48	28

24	44	TestSample	30.08	0.64	
25	45	TestSample	30.25	0.47	
26	46	TestSample	28.00	2.72	29
27	47	TestSample	29.60	1.12	
28	48	TestSample	30.09	0.63	
29	49	TestSample	30.32	0.40	
30	50	TestSample	30.72	0.00	
31	51	TestSample	30.30	0.42	
32	52	TestSample	30.53	0.19	
33	53	TestSample	30.50	0.22	
34	54	TestSample	30.41	0.31	
35	55	TestSample	30.02	0.70	
36	56	TestSample	31.01	-0.29	
37	57	TestSample	29.93	0.79	
38	58	TestSample	29.48	1.24	
39	59	TestSample	30.05	0.67	
40	60	TestSample	30.60	0.12	
41	61	TestSample	30.59	0.13	
42	62	TestSample	29.71	1.01	
43	63	TestSample	29.78	0.94	
44	64	TestSample	29.85	0.87	
45	65	TestSample	30.42	0.30	
46	66	TestSample	30.82	-0.10	
47	67	TestSample	30.08	0.64	
49	69	TestSample	30.28	0.44	
51	71	TestSample	28.03	2.69	30
52	72	TestSample	29.17	1.55	31
53	73	TestSample	29.01	1.71	32
54	74	TestSample	30.45	0.27	
55	75	TestSample	30.06	0.66	
56	76	TestSample	29.06	1.66	33
57	77	TestSample	30.22	0.50	
58	78	TestSample	31.12	-0.40	
59	79	TestSample	30.48	0.24	
60	80	TestSample	30.51	0.21	
61	81	TestSample	29.93	0.79	
62	82	TestSample	30.53	0.19	
63	83	TestSample	28.11	2.61	34
64	84	TestSample	29.21	1.51	35
65	85	TestSample	29.29	1.43	
66	86	TestSample	30.02	0.70	
67	87	TestSample	29.71	1.01	
68	MP/Actiphage	NegativeControl	30.72	n/a	
69	MAP -2	PositiveControl	22.18	n/a	
70	Kit Control EPC	PositiveControl	19.07	n/a	

No.	63
Total	203
week 3	

Second Run			Green MAP	ΔCq	
Position	Sample name	Sample type	Cq Value		
1	Set1: 21	TestSample	31.14	0.38	
2	Set1: 22	TestSample	32.23	-0.71	
3	Set1: 21	TestSample	31.51	0.01	
4	Set1: 22	TestSample	31.00	0.52	
5	Set1: 23	TestSample	31.91	-0.39	
6	Set1: 24	TestSample	29.93	1.59	
7	Set 1: 67	TestSample	28.36	3.16	36
8	Set 1: 68	TestSample	31.15	0.37	
9	Set 1: 69	TestSample	30.12	1.40	
10	Set 1: 70	TestSample	30.83	0.69	
11	Set 1: 71	TestSample	30.56	0.96	
12	Set 1: 72	TestSample	30.41	1.11	
13	Set 1: 73	TestSample	31.48	0.04	
14	Set 1: 74	TestSample	30.52	1.00	
15	Set 1: 75	TestSample	30.53	0.99	
16	MP/Actiphage	NegativeControl	31.52	n/a	
17	MAP -2	PositiveControl	23.89	n/a	
18	Kit Control EPC	PositiveControl	20.68	n/a	

No.	15
Total	218

week 4

First Run			Green MAP	ΔCq
Position	Sample name	Sample type	Cq Value	
31	Pg 77 Lab ID: 1	TestSample	30.75	1.69
32	Pg 77 Lab ID: 2	TestSample	30.84	1.60
33	Pg 77 Lab ID: 3	TestSample	31.25	1.19
34	Pg 77 Lab ID: 4	TestSample	32.76	-0.32
35	Pg 77 Lab ID: 5	TestSample	31.82	0.62
36	Pg 77 Lab ID: 6	TestSample	31.28	1.16
37	Pg 77 Lab ID: 7	TestSample	32.09	0.35
38	Pg 77 Lab ID: 8	TestSample	31.99	0.45
39	Pg 77 Lab ID: 9	TestSample	31.45	0.99
40	Pg 77 Lab ID: 10	TestSample	31.71	0.73
41	Pg 77 Lab ID: 11	TestSample	31.67	0.77
42	Pg 77 Lab ID: 12	TestSample	31.66	0.78
43	Pg 77 Lab ID: 13	TestSample	31.11	1.33
44	Pg 77 Lab ID: 14	TestSample	32.47	-0.03
45	Pg 77 Lab ID: 15	TestSample	31.72	0.72
46	Pg 77 Lab ID: 16	TestSample	32.06	0.38
47	Pg 77 Lab ID: 17	TestSample	31.92	0.52
48	Pg 77 Lab ID: 18	TestSample	32.33	0.11
49	Pg 77 Lab ID: 19	TestSample	31.71	0.73
50	Pg 77 Lab ID: 20	TestSample	31.57	0.87
51	Pg 77 Lab ID: 21	TestSample	31.08	1.36

52	Pg 77 Lab ID: 22	TestSample	31.36	1.08
53	Pg 77 Lab ID: 23	TestSample	31.49	0.95
54	Pg 77 Lab ID: 24	TestSample	24.14	8.30
55	Pg 77 Lab ID: 25	TestSample	31.15	1.29
56	Pg 77 Lab ID: 26	TestSample	31.29	1.15
57	Pg 77 Lab ID: 27	TestSample	31.58	0.86
58	Pg 77 Lab ID: 28	TestSample	32.03	0.41
59	Pg 77 Lab ID: 29	TestSample	32.42	0.02
60	Pg 77 Lab ID: 30	TestSample	31.67	0.77
61	Pg 77 Lab ID: 31	TestSample	31.61	0.83
62	Pg 77 Lab ID: 32	TestSample	28.30	4.14
63	Pg 77 Lab ID: 33	TestSample	32.13	0.31
64	Pg 77 Lab ID: 34	TestSample	32.11	0.33
65	Pg 77 Lab ID: 35	TestSample	32.89	-0.45
66	Pg 77 Lab ID: 36	TestSample	30.59	1.85
67	Pg 77 Lab ID:37	TestSample	31.81	0.63
68	Pg 77 Lab ID:38	TestSample	32.71	-0.27
69	MP/Actiphage	NegativeControl	32.44	n/a
70	MAP -2 Pg 77	PositiveControl	18.68	n/a

No. 38
Total 256

**Second Run**			**Green MAP**	**ΔCq**	
**Position**	**Sample name**	**Sample type**	**Cq Value**		
1	Lab ID: 39	TestSample	37.21	-4.25	
2	Lab ID: 40	TestSample	35.24	-2.28	
3	Lab ID: 41	TestSample	34.30	-1.34	
4	Lab ID: 42	TestSample	32.81	0.15	
5	Lab ID: 43	TestSample	34.84	-1.88	
6	Lab ID: 44	TestSample	35.18	-2.22	
7	Lab ID: 45	TestSample	36.54	-3.58	
8	Lab ID: 46	TestSample	36.06	-3.10	
9	Lab ID: 47	TestSample	36.34	-3.38	
10	Lab ID: 48	TestSample	35.20	-2.24	
11	Lab ID: 49	TestSample	31.37	1.59	41
12	Lab ID: 50	TestSample	31.05	1.91	43
13	Lab ID: 51	TestSample	30.72	2.24	44
14	Lab ID: 52	TestSample	30.95	2.01	45
15	Lab ID: 53	TestSample	31.04	1.92	46
16	Lab ID: 54	TestSample	34.29	-1.33	
17	Lab ID: 55	TestSample	31.49	1.47	
18	Lab ID: 56	TestSample	30.63	2.33	47
19	Lab ID: 57	TestSample	35.17	-2.21	
20	Lab ID: 58	TestSample	32.00	0.96	
21	Lab ID: 59	TestSample	34.83	-1.87	
22	Lab ID: 60	TestSample	34.36	-1.40	
23	Lab ID: 61	TestSample	34.33	-1.37	

24	Lab ID: 62	TestSample	34.14	-1.18	
25	Lab ID: 63	TestSample	34.16	-1.20	
26	Lab ID: 64	TestSample	34.34	-1.38	
27	Lab ID: 65	TestSample	35.47	-2.51	
28	Lab ID: 66	TestSample	31.81	1.15	
29	Lab ID: 67	TestSample	34.11	-1.15	
30	Lab ID: 68	TestSample	35.27	-2.31	
31	Lab ID: 69	TestSample	35.25	-2.29	
32	Lab ID: 70	TestSample	32.86	0.10	
33	Lab ID: 71	TestSample	33.30	-0.34	
34	Lab ID: 72	TestSample	32.18	0.78	
35	Lab ID: 73	TestSample	31.93	1.03	
36	Lab ID: 74	TestSample	31.75	1.21	
37	Lab ID: 75	TestSample	33.84	-0.88	
38	Lab ID: 76	TestSample	33.15	-0.19	
39	Lab ID: 77	TestSample	33.63	-0.67	
40	Lab ID: 78	TestSample	33.20	-0.24	
41	Lab ID: 79	TestSample	32.25	0.71	
42	Lab ID: 80	TestSample	30.80	2.16	48
43	MAP -2	PositiveControl	20.97	n/a	
44	MP/Actiphage	NegativeControl	32.96	n/a	
45	Kit Contro EPC	PositiveControl	20.41	n/a	

No.	42	% positive
Total	298	16.1
